# Implantable aptamer–field-effect transistor neuroprobes for in vivo neurotransmitter monitoring

**DOI:** 10.1126/sciadv.abj7422

**Published:** 2021-11-24

**Authors:** Chuanzhen Zhao, Kevin M. Cheung, I-Wen Huang, Hongyan Yang, Nako Nakatsuka, Wenfei Liu, Yan Cao, Tianxing Man, Paul S. Weiss, Harold G. Monbouquette, Anne M. Andrews

**Affiliations:** 1 Department of Chemistry and Biochemistry, University of California, Los Angeles, Los Angeles, CA 90095, USA.; 2 California NanoSystems Institute, University of California, Los Angeles, Los Angeles, CA 90095, USA.; 3 Department of Chemical and Biomolecular Engineering, University of California, Los Angeles, Los Angeles, CA 90095, USA.; 4 Department of Psychiatry and Biobehavioral Sciences, Semel Institute for Neuroscience and Human Behavior, Hatos Center for Neuropharmacology, University of California, Los Angeles, Los Angeles, CA 90095, USA.; 5 Department of Mechanical and Aerospace Engineering, University of California, Los Angeles, Los Angeles, CA 90095, USA.; 6 Department of Materials Science and Engineering, University of California, Los Angeles, Los Angeles, CA 90095, USA.; 7 Department of Bioengineering, University of California, Los Angeles, Los Angeles, CA 90095, USA.

## Abstract

While tools for monitoring in vivo electrophysiology have been extensively developed, neurochemical recording technologies remain limited. Nevertheless, chemical communication via neurotransmitters plays central roles in brain information processing. We developed implantable aptamer–field-effect transistor (FET) neuroprobes for monitoring neurotransmitters. Neuroprobes were fabricated using high-throughput microelectromechanical system (MEMS) technologies, where 150 probes with shanks of either 150- or 50-μm widths and thicknesses were fabricated on 4-inch Si wafers. Nanoscale FETs with ultrathin (~3 to 4 nm) In_2_O_3_ semiconductor films were prepared using sol-gel processing. The In_2_O_3_ surfaces were coupled with synthetic oligonucleotide receptors (aptamers) to recognize and to detect the neurotransmitter serotonin. Aptamer-FET neuroprobes enabled femtomolar serotonin detection limits in brain tissue with minimal biofouling. Stimulated serotonin release was detected in vivo. This study opens opportunities for integrated neural activity recordings at high spatiotemporal resolution by combining these aptamer-FET sensors with other types of Si-based implantable probes to advance our understanding of brain function.

## Teaser

Implantable aptamer transistor probes for in vivo neurotransmitter monitoring advance brain activity recording.

## INTRODUCTION

Determining how information is encoded in brain function is at the heart of neuroscience. Discoveries in brain information processing lead to improved understanding of healthy brain function and the etiologies of neurological and neuropsychiatric disorders (*1*–*6*). Decoding neural function requires advanced technologies to make multiplexed measurements that approach the spatial scales and temporal dynamics of chemical neurotransmission. Implantable neural recording probes have emerged as powerful tools to monitor brain activity with high spatiotemporal resolution (*7*–*9*). Efforts in implantable electrode development have focused on monitoring electrical signals, which can be sorted and analyzed to decode spiking activity in single neurons (*10*, *11*). With recent innovations in micro- and nanofabrication, materials science, surface functionalization, and electrical engineering, implantable neural devices have become more sophisticated so as to have increased densities of recording elements with reduced sizes (*12*–*19*). Recently, up to ~1000 electrophysiological recording units have been integrated into single neuroprobes of <100-μm width (*16*, *20*).

We developed aptamer–field-effect transistor (FET) biosensors for electronic small-molecule detection under high ionic strength conditions (*21*–*23*). We used nanoscale In_2_O_3_ semiconducting films (3 to 4 nm) as an ultrasensitive platform for biosensing. Aptamers selected for specific target recognition were coupled to the semiconductor surfaces of FETs (*23*). Conformational changes of the negatively charged aptamer backbones occur upon target capture. The subsequent surface charge redistribution is detected by the voltage-gated semiconductor. This sensing mechanism is independent of the charge or electrochemical properties of the analytes themselves and thus represents a universal approach for monitoring small molecules (*23*).

We detected a number of different targets in complex physiological environments using aptamer FETs, including the neurotransmitters serotonin and dopamine, glucose, and the amino acid phenylalanine (*21*–*24*). Because of the high selectivity of the aptamers we use, aptamer-FET biosensors recognize their specific targets but not structurally similar molecules. We recently reported the real-time and simultaneous detection of serotonin and dopamine using aptamer-FET biosensor arrays, establishing a foundation for multiplexed monitoring of brain neurotransmitters and other targets (*21*).

**Fig. 1.**
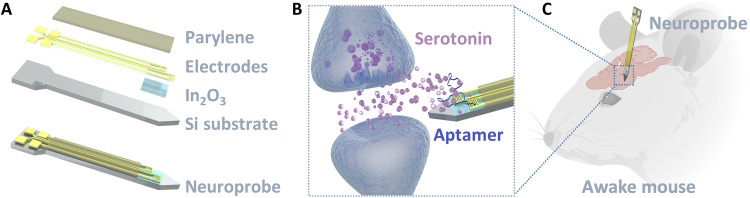
Schematic illustrations of the design and application of implantable aptamer-FET neuroprobes. (**A**) Layer-by-layer design of a neuroprobe with two FETs at the tip. Top to bottom: Parylene, Au electrodes, In_2_O_3_, the Si substrate, and a fully constructed neuroprobe. (**B**) Illustration showing released serotonin in the extracellular space monitored by an aptamer-FET neuroprobe (not to scale). (**C**) Illustration of a neuroprobe implanted in the brain of a mouse for in vivo neurotransmitter monitoring.

Here, we advance our approach by designing, fabricating, and testing implantable aptamer-FET neuroprobes to monitor the small-molecule neurotransmitter serotonin. We investigated device functionality in vitro, ex vivo, and in vivo (scheme shown in Fig. 1). We designed Si-based neuroprobes with In_2_O_3_ FETs on the shank tips (Fig. 1, A and B). Serotonin aptamers were functionalized on the In_2_O_3_ surfaces of the FETs to detect serotonin. We show that aptamer-FET neuroprobes can monitor serotonin flux in vivo in real time.

## RESULTS

**Fig. 2.**
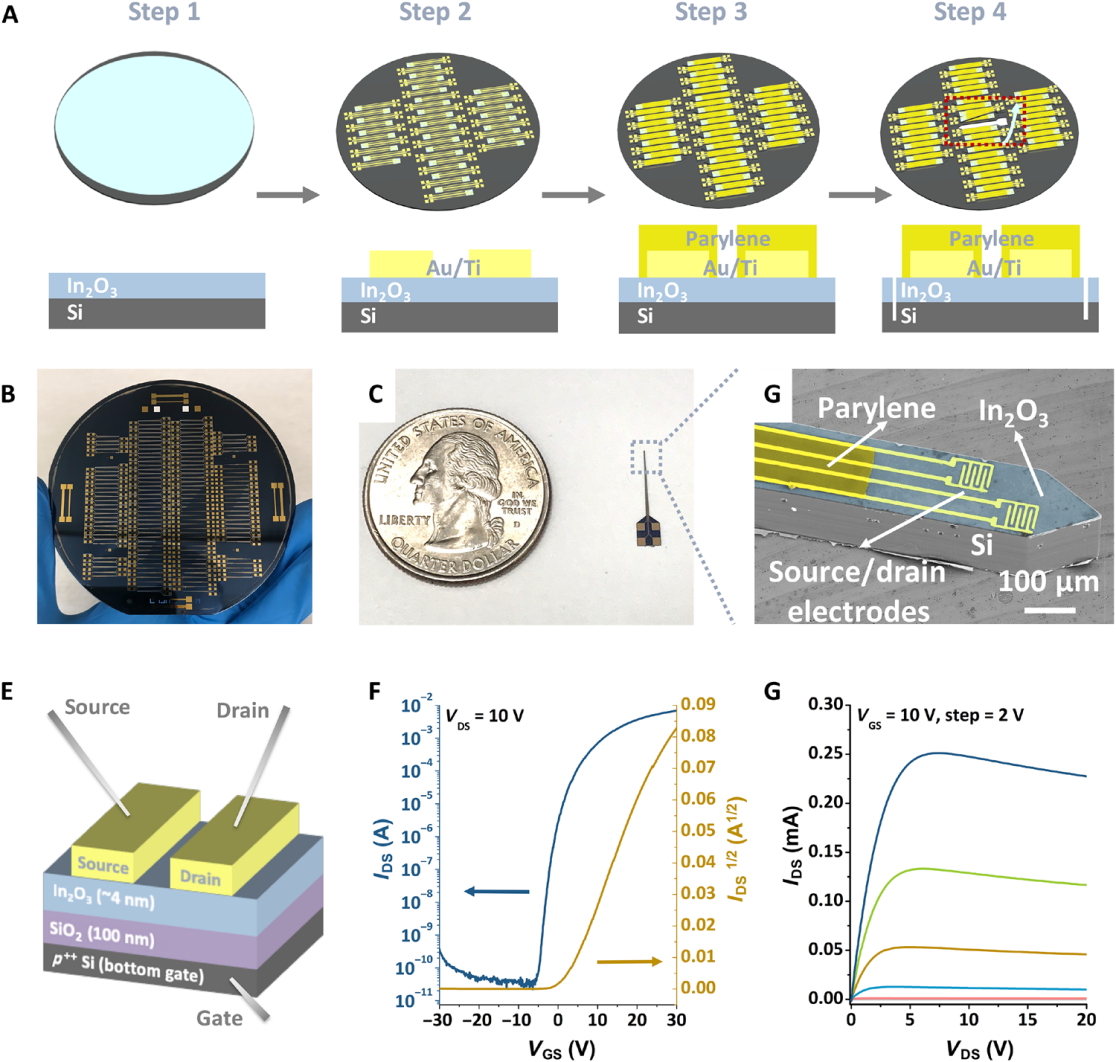
Neuroprobe fabrication and FET characterization. (**A**) Schematic illustration showing the neuroprobe fabrication process. (**B**) Photograph of a 4-inch Si wafer with 150 fabricated 150-μm neuroprobes after deep reactive-ion etching but before individual probe release. (**C**) Photograph showing a released 150-μm-wide by 150-μm-thick neuroprobe next to a U.S. quarter dollar coin to illustrate neuroprobe size. (**D**) Scanning electron microscope (SEM) image of the shank and tip of a 150-μm-wide by 150-μm-thick neuroprobe with two staggered FETs. False colors show the parylene layer, Au electrodes, In_2_O_3_, and the Si substrate. (**E**) Schematic illustration of the solid-state measurement setup (layers not to scale). (**F** and **G**) Representative transfer and output characteristics for 60 μm–by–80 μm FETs, respectively. Photo credit: Chuanzhen Zhao, UCLA.

A schematic illustration of the neuroprobe fabrication process is shown in Fig. 2A. Here, microelectromechanical system (MEMS) technologies were used to produce neuroprobes in a high-throughput manner, where 150 probes were fabricated on each Si wafer. This fabrication process is compatible with conventional microfabrication processes, which is advantageous for integrating additional sensors and actuators previously fabricated on Si or other biomaterials (e.g., temperature, enzyme-based, photonic, and electrophysiology sensors, and optical and microfluidic actuators) (*14*, *16*, *25*–*27*). The MEMS fabrication approach enables the production of large numbers of devices needed for translation to neuroscience applications.

Briefly, thin films of In_2_O_3_ were formed via sol-gel chemistry (*28*) by spin-coating an aqueous solution of indium(III) nitrate hydrate onto heavily doped 4-inch Si wafers (150-μm-thick *p*^++^ Si with 100-nm-thick SiO_2_ on top) (step 1 in Fig. 2A). We used In_2_O_3_ as the semiconductor because of the straightforward fabrication of thin (~3 to 4 nm) layers that impart high sensitivity associated with high surface-to-volume ratios and stable performance in electrolyte solutions compared with other metal oxides [e.g., indium-gallium-zinc oxide (IGZO) or ZnO] (*29*–*31*). The Au and Ti electrodes (30 and 10 nm thick, respectively) were then patterned on top of the In_2_O_3_ (step 2 in Fig. 2A). In some cases, a thin layer of parylene (~1 μm) was coated on the probe surfaces to provide insulation of the interconnects (step 3 in Fig. 2A). Parylene is a biocompatible and biostable material with excellent dielectric properties that is widely used in implanted medical devices, such as cardiac assist devices and mandrel catheters (*32*, *33*). The parylene layer at the tip of each probe shank was removed to expose the transistor area for sensing. Outlines of individual probes were defined using an additional photolithography step. Wafers were then etched through to release the probes (step 4 in Fig. 2A). The fabrication process is described in detail in the Materials and Methods.

A 4-inch wafer with 150 probes is shown before the probe release step in Fig. 2B. The entire wafer was semitransparent after the etching process used to define the probe outlines. A single probe is shown after release in Fig. 2C. The neuroprobe shanks were characterized using scanning electron microscopy (SEM; Fig. 2D). Pseudo-colors were assigned to different components, where parylene (dark yellow) is the insulating layer. The Au and Ti source and drain electrodes (yellow) were constructed in an interdigitated design to increase the channel width for higher sensitivity (*23*). The exposed Au in the interdigitated electrode region was insulated by an alkanethiol self-assembled monolayer, as described in the Materials and Methods. The In_2_O_3_ (light blue, thin film without patterning in this fabrication process) was coated onto each 150-μm-thick Si wafer (gray). Each probe had an overall width of 150 μm, and two FETs (60 μm by 80 μm) were separated by 20 μm along the probe length and 10 μm across the probe width.

Solid-state measurements were carried out to test FET performance in a bottom-gate top-contact configuration (Fig. 2E). Representative transfer and output characteristics are shown in Fig. 2, F and G, respectively. Each FET measured 80 μm by 60 μm, and the FET channel width/length was 200 μm/5 μm. These miniaturized FETs showed high current on/off ratios (*I*_on_/*I*_off_) of ~10^8^, comparable to our devices with millimeter dimensions (*23*).

**Fig. 3.**
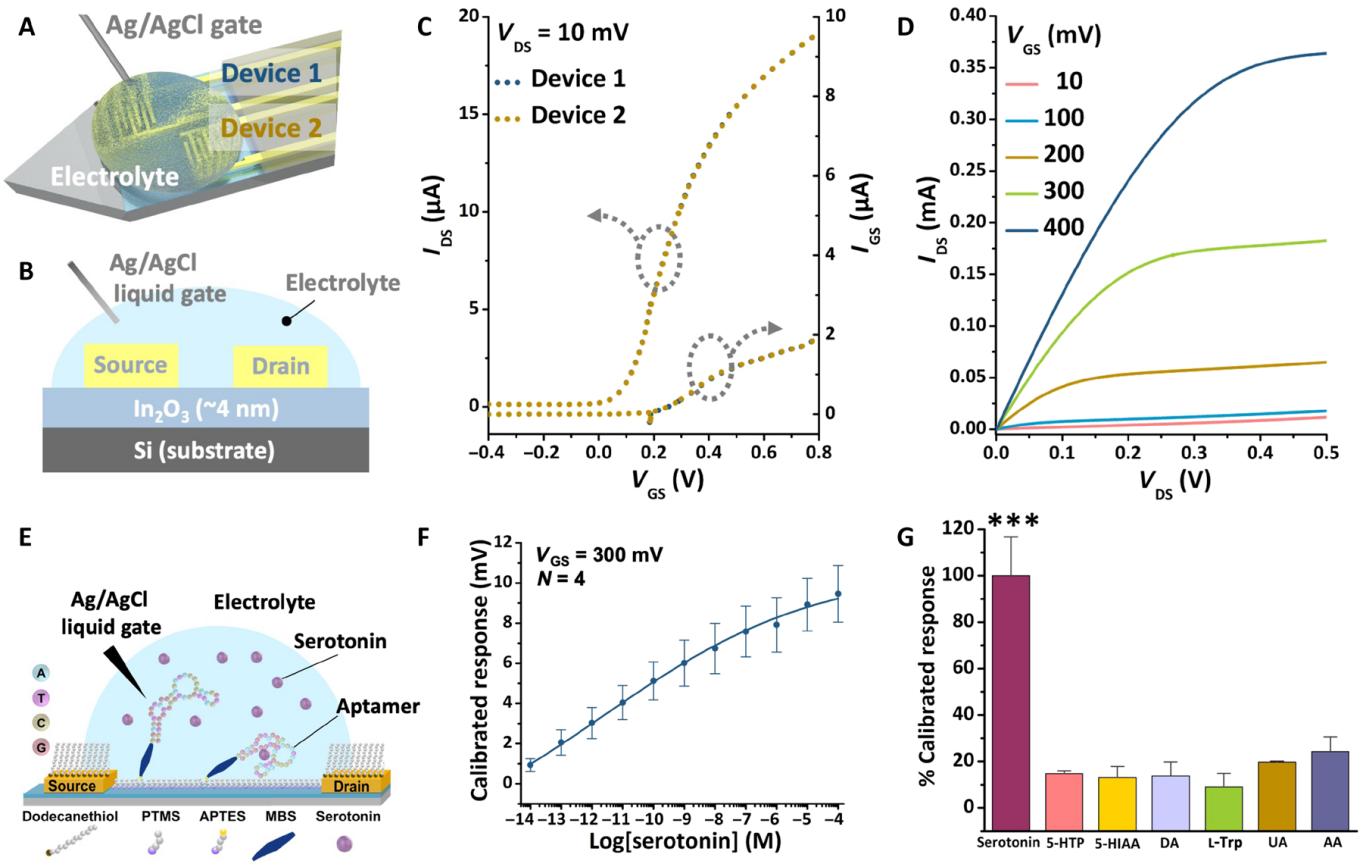
Neuroprobe biosensing in vitro. (**A** and **B**) Schematic illustrations of the liquid-gate measurement setup [layers not to scale in (B)]. (**C**) Representative transfer characteristics (*I*_DS_-*V*_GS_; left) and leakage current (*I*_GS_-*V*_GS_; right) for two transistors (curves are overlaid) on a single probe in phosphate-buffered saline. (**D**) Representative transfer characteristics (*I*_DS_-*V*_DS_) at different gate voltages showing typical transistor behavior with saturation. (**E**) Schematic illustration showing the surface functionalization for In_2_O_3_ transistor channels. PTMS, trimethoxy(propyl)silane; APTES, (3-aminopropyl)triethoxysilane; MBS, 3-maleimidobenzoic acid *N*-hydroxysuccinimide ester. (**F**) Serotonin aptamer-FET response curve in artificial cerebrospinal fluid (aCSF). Error bars are SEM from *N* = 4 FETs. (**G**) Serotonin aptamer-functionalized neuroprobe responses to biologically relevant concentrations of interferents versus serotonin in aCSF (100 nM): 100 μM l-5-hydroxytryptophan (l-5-HTP), 5-hydroxyindoleacetic acid (5-HIAA), dopamine (DA), l-tryptophan (l-Trp), 50 μM uric acid (UA), or 200 μM ascorbic acid (AA). Error bars are standard errors of the means for *N* = 4 FETs for serotonin and *N* = 3 FETs for nontarget molecules. ***P < 0.005 versus nontargets.

For biosensing, FET-based neuroprobes were operated in electrolyte solutions via liquid gating. The FETs in electrolyte solutions showed improved transistor characteristics because of the high dielectric constant of physiological solutions (see equation and detailed explanation in the Supplementary Materials) (*23*). Two FETs were fabricated in a staggered configuration on the tip of each neuroprobe and were gated by a Ag/AgCl reference electrode (Fig. 3A). A schematic of the liquid-gate transistor operation setup is shown in Fig. 3B. Here, the electrical double layer formed in the electrolyte solution serves as the gate dielectric. Representative transfer and output characteristics are shown in Fig. 3, C and D, respectively. The transfer curves of two different devices overlapped with minimal contribution from gate leakage currents (Fig. 3C).

To construct biosensors, thiol-terminated DNA aptamers were immobilized onto In_2_O_3_ surfaces using (3-aminopropyl)triethoxysilane and 3-maleimidobenzoic acid *N-*hydroxysuccinimide ester for linking (Fig. 3E) (*21*, *23*). In vitro serotonin detection was performed in artificial cerebrospinal fluid (aCSF), which mimics the ionic strength and composition of the brain extracellular fluid (*34*). Aptamer-FET neuroprobes detected serotonin over a large concentration range (fM to μM; Fig. 3F). We used calibrated responses to minimize device-to-device variations [information on calculations is provided in the Supplementary Materials (*23*, *35*)]. The serotonin detection range on neuroprobes with micrometer-scale FETs was similar to that previously reported for FET sensors with mm^2^ dimensions because of the quasi–two-dimensional In_2_O_3_ semiconductor channels used in the design of FETs of both sizes (*23*).

Neuroprobes were selective in detecting serotonin with respect to serotonin precursors (i.e., tryptophan and l-5-hydroxytryptophan), the major serotonin metabolite 5-hydroxyindoleacetic acid, the monoamine neurotransmitter dopamine, and uric acid and ascorbic acid, all of which coexist in extracellular fluid (Fig. 3G; see table S1 for statistics). Some of these species are present in brain extracellular fluid at 1000× greater concentrations than serotonin; all are potential interferents during in vivo sensing (*23*, *36*, *37*). The selectivity of aptamer-FET neuroprobes is intrinsic to the aptamers. For other neurochemical sensing platforms, such as fast-scan cyclic voltammetry or enzyme-based neural probes, nonspecific signals generated via the oxidation or reduction of interfering electroactive species or nonspecific H_2_O_2_ production, respectively, can complicate target-specific and multiplexed neurotransmitter detection (*38*).

**Fig. 4.**
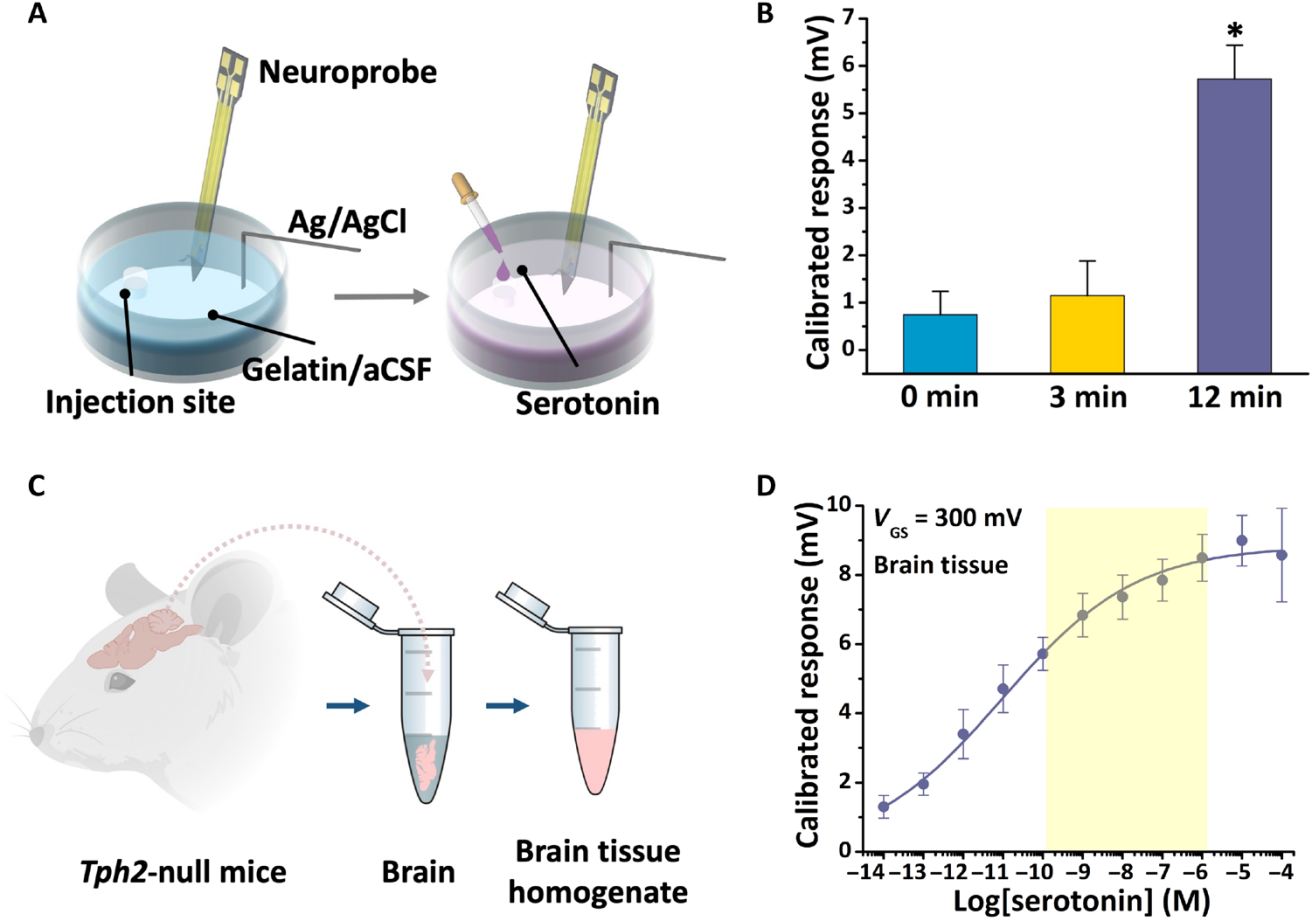
In vitro and ex vivo neuroprobe serotonin sensing. (**A**) Schematic illustrations showing an in vitro experiment in a brain-mimicking solid matrix composed of gelatin in aCSF. (**B**) Calibrated responses after the addition of 100 nM serotonin over time. Error bars are standard errors of the means for *N* = 2 individual probes. *P < 0.05 versus 0- and 3-min time points. (**C**) Schematic illustration of the preparation of a brain tissue homogenate. Brains from *Tph2*-null mice were removed, and tissue was homogenized in aCSF. (**D**) Serotonin aptamer-FET response curve in brain tissue homogenates. The highlighted region represents serotonin concentrations in the extracellular space in vivo. Error bars are standard errors of the means for *N* = 3 individual FETs.

Brain tissue is a complex biological matrix. Neurotransmitters diffuse from release sites through tissue to probe recording sites over at least a couple hundred micrometers. To test the function of neuroprobes in solid matrices that mimic the tortuosity of brain tissue, we used 10 to 15% gelatin in aCSF to mimic the Young’s modulus and stiffness (*39*) and physiological ionic environment of brain tissue. Gelatin was cast in 48-well plates, where each well mimics the size of a mouse brain (~1 cm in diameter). As shown in fig. S1, a hole was templated into each gelatin mold for the addition of serotonin (100 nM) to simulate neurotransmitter release and diffusion in the brain (Fig. 4A). This concentration was chosen to represent a physiologically relevant serotonin concentration (*34*, *36*, *40*–*42*).

Data were collected immediately after the introduction of serotonin, and 3 and 12 min later (Fig. 4B). There were significant increases in FET responses 12 min after serotonin addition (see table S1 for statistics), demonstrating that the aptamer-FET neuroprobes detected diffusion-related changes in serotonin levels with respect to time. We previously found that the response times of our aptamer-FET sensors are on the order of seconds (*21*, *23*); the 12-min response time here is due to target diffusion over ~0.2 cm in the gelatin/aCSF matrix from the addition location to the recording site.

We tested neuroprobes in brain tissue ex vivo to assess their ability to operate in a complex biological matrix that more closely approximates the in vivo environment (fig. S2). Brain tissue from *Tph2*-null mice lacking expression of the rate-limiting serotonin synthetic enzyme in the central nervous system (CNS) (*43*) was homogenized in aCSF to provide a tissue environment devoid of endogenous serotonin (Fig. 4C). As shown in Fig. 4D, serotonin added exogenously was detected via aptamer-FET neuroprobes over a concentration range similar to that recorded in aCSF (i.e., fM to μM), indicating that biofouling occurring during the measurement period of ~1 hour did not interfere with serotonin detection. The in vivo concentration of extracellular serotonin is highlighted (*34*, *36*, *40*–*42*), showing that the detection range of the aptamer neuroprobes in brain tissue covered the estimated extracellular serotonin concentration range (Fig. 4D). The sensitivity and selectivity of the aptamer neuroprobes, in addition to the results from the gelatin and brain tissue homogenate experiments, illustrated the potential for in vivo studies.

To evaluate the in vivo capability of the neuroprobes, a sensing experiment was performed using electrical stimulation to release serotonin. Electrical stimulation is widely used in vivo to induce neurotransmitter release in the CNS (*44*). However, as the detection mechanism of FET-based biosensors relies on aptamer and solution-ion charge redistribution near the semiconductor surface, external electrical stimulation could affect FET signals associated with target capture. To test for electrical interference, neuroprobes were placed in phosphate-buffered saline (PBS) with stimulating electrodes, as shown in fig. S3 (A and B). The *I*_DS_-*V*_GS_ sweeps were collected before and after electrical stimulation (biphasic pulses of 300 μA, pulse width of 4 ms, and 30 Hz for 5 s; fig. S3C). The FET measurements after stimulation showed negligible differences compared to measurements before stimulation, suggesting little influence of the electrical stimulation on signals measured from the aptamer-FET neuroprobes. The different time scales for neurochemical versus endogenous electrophysiological events likely preclude our sensors, with temporal resolution on the order of seconds, from nonspecifically detecting neurotransmitter-evoked changes in local field potentials (~100 ms).

**Fig. 5.**
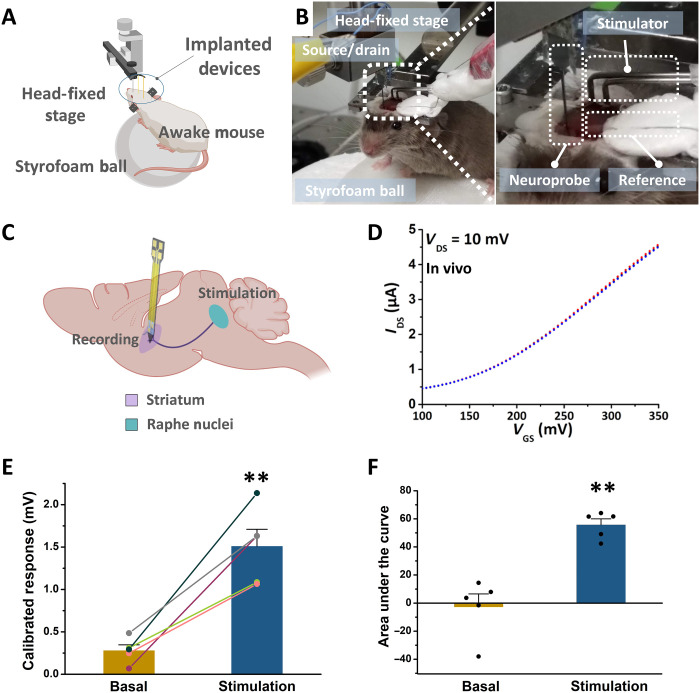
In vivo serotonin monitoring using an aptamer-FET neuroprobe. (**A**) Schematic illustration and (**B**) photographs of an in vivo experiment, where the neuroprobe, Ag/AgCl reference electrode, and stimulator were implanted into the brain of a head-fixed mouse. (**C**) Schematic illustration of stimulation and recording sites. The stimulating electrode was implanted into the serotonin cell-body region, and the neuroprobe was implanted into a serotonin terminal region in the striatum. (**D**) Three consecutive overlapping output sweeps (*I*_DS_-*V*_GS_) in vivo, where *V*_GS_ was swept from 100 to 350 mV, while *V*_DS_ was held at constant 10 mV. (**E**) Calibrated responses and (**F**) areas under the curves for in vivo determination of basal and post-electrical stimulation levels from the same mouse, respectively (*V*_GS_ = 300 mV). Error bars in E. and F. are standard errors of the means. **P < 0.01 versus basal. Photo credit: Kevin M. Cheung, UCLA.

An in vivo experiment was conducted in a female mouse that constitutively lacked serotonin transporter expression. The serotonin transporter takes up serotonin from the extracellular space. Mice that do not express this transporter have higher basal and stimulated serotonin levels (*34*, *41*). The mouse was acclimated over a number of days of behavior training to being head-fixed (*5*, *45*). A schematic of the experiment is shown in Fig. 5A. Photographs of the experiment are shown in Fig. 5B, where a neuroprobe, Ag/AgCl reference electrode, and stimulating electrode are shown implanted into the brain of the mouse. The stimulating electrode was located just above the brain stem serotonin cell bodies. The neuroprobe was implanted into the striatum where serotonin axons project (*41*). Electrical stimulation of serotonin cell bodies releases serotonin in the striatum (Fig. 5C). Detailed information is included in the Materials and Methods.

After implantation, we tested device functionality by collecting transfer curves (*I*_DS_-*V*_GS_) from the FETs. As shown in Fig. 5D, the transfer curves showed typical FET characteristics. Three overlapping transfer curves were collected immediately before electrical stimulation, showing that the biosensors were relatively stable when implanted in the brain without observable drift over short times. For in vivo serotonin measurements, prestimulus measurements were made followed by electrical stimulation and determination of serotonin release. Increases in FET-calibrated responses after electrical stimulation were observed, indicating that the neuroprobe having a FET functionalized with a serotonin-specific aptamer detected increases in serotonin after stimulation (Fig. 5, E and F, and fig. S4). Representative continuous calibrated responses are shown in fig. S4 for the 1-min period before stimulation (basal levels) and 1 min after stimulation. As summarized in Fig. 5 (E and F), aptamer-FET biosensors differentiated basal and stimulated serotonin levels, suggesting minimal sensor drift over the measurement period. We previously carried out continuous real-time *I*_DS_ monitoring under constant voltage bias, where aptamer FETs showed stable signals over 20 min with little drift (*21*). Here, the temporal resolution is limited by the measurement instrument, which takes ~5 s for each gate voltage sweep. We previously showed ~2-s temporal resolution in real-time *I*_DS_ measurements (*21*, *23*).

In mice lacking the serotonin transporter, clearance of extracellular serotonin depends on diffusion and uptake by low-affinity transporters (e.g., dopamine, plasma membrane monoamine, or organic cation transporters) (*46*–*48*). Thus, the clearance time of extracellular serotonin is prolonged in mice lacking high-affinity serotonin uptake (*49*, *50*). This slower clearance process (fig. S4) is consistent with the time course of electrically stimulated serotonin release after pharmacologic inhibition of serotonin transporters determined by fast cyclic square-wave voltammetry (*36*).

**Fig. 6.**
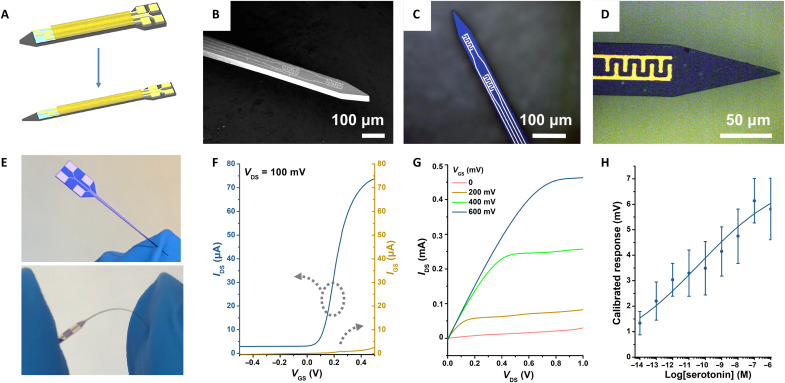
Fabrication and characterization of flexible 50-μm neuroprobes. (**A**) Schematic illustration comparing scaling between a 150-μm-thick and 150-μm-wide probe and a 50-μm-thick and 50-μm-wide probe. (**B**) SEM image of the shank and tip of a 50-μm neuroprobe showing a two-FET configuration. (**C** and **D**) Optical microscope images of 50-μm probes. (**E**) Photographs showing a 50-μm neuroprobe, which is stiff enough to penetrate a nitrile glove (top) yet can be easily bent (bottom). (**F** and **G**) Representative transfer and output characteristics in aCSF, respectively. (**H**) Serotonin aptamer-FET response curve in aCSF. Error bars are standard errors of the means for *N* = 4 individual 30 μm–by–50 μm FETs. Photo credit: Chuanzhen Zhao, UCLA.

The 150-μm Si probes showed promising in vivo results. Nonetheless, rigid Si-based devices are less desirable for long-term recordings because of the inflammatory responses they evoke (*51*–*53*). One strategy to improve the biomaterial interface is to reduce stiffness by fabricating devices with reduced dimensions. The bending stiffness *D* is defined as

$$D\propto {\mathrm{Et}}^{3}$$

where *E* is the Young’s modulus and *t* is the thickness. Stiffness scales cubically with device thickness (*52*–*54*). Hence, we designed second-generation Si neuroprobes with dimensions reduced by two-thirds (i.e., 50-μm thick and 50-μm wide) (Fig. 6A).

The fabrication process for the 50-μm neuroprobes was similar to the fabrication of 150-μm probes except that a thinner 50-μm Si wafer was used for the former. The smaller probes are shown in Fig. 6 (B to E), where two transistors (30 μm by 50 μm each) were patterned on the tip of each probe. The Si probes with reduced size maintained sufficient penetration stiffness yet showed flexibility (Fig. 6E and movie S1). We previously demonstrated that flexible In_2_O_3_ FETs are stable even after many bending or crumpling cycles with minimal changes in mobility and device performance (*21*). The smaller FETs on the 50-μm probes showed typical transistor performance indicated by their transfer and output curves (Fig. 6, F and G, respectively). We functionalized these smaller FETs with serotonin aptamers. Similar to larger aptamer FETs, the smaller versions showed serotonin sensitivity down to femtomolar concentrations.

## DISCUSSION

We developed implantable aptamer-FET neuroprobes for in vivo neurotransmitter detection. Ultrathin In_2_O_3_ (~3 to 4 nm) with a high surface-to-volume ratio was prepared using a sol-gel process and used as the channel material in FETs. High-throughput MEMS technologies were used to fabricate 150 neuroprobes per 4-inch Si wafer, where each probe was 150 or 50 μm wide and thick at the shank. Aptamers were coupled to In_2_O_3_ surfaces to achieve selective detection of serotonin in the micromolar to femtomolar range in physiological environments (i.e., aCSF and brain tissue homogenates). We showed that neuroprobes were functional in vitro in gelatin/aCSF, a material that simulates a tissue matrix, in brain tissue, and under electrical stimulation conditions used to stimulate serotonin release in vivo.

The neuroprobes described represent a general strategy for monitoring neurochemicals in the brain. Previously, we showed that, in addition to serotonin, aptamer FET biosensors can be used to detect dopamine, glucose, sphingosine-1-phosphate, and phenylalanine. Aptamers for other neurotransmitters have been reported (*55*, *56*), and new, highly selective aptamers are being developed (e.g., norepinephrine, epinephrine, glutamate, γ-aminobutyric acid, and dynorphin). As we continue to identify and to characterize additional aptamers, we envision that our approach will enable multiplexed monitoring of many different neurotransmitters in the brain with high sensitivity and selectivity, regardless of their electroactivity or redox enzymes available for them. We briefly highlight the advantages of aptamer FET biosensors compared to other currently used neurochemical recording and imaging techniques in the Supplementary Materials (table S2).

Aptamer biosensors can be multiplexed because of the high selectivity of our aptamers and our ability to fabricate FETs at small scales and high densities (*30*). We recently developed multiplexed sensor arrays to monitor serotonin, dopamine, pH, and temperature simultaneously in real time (*21*). The capability to monitor multiple neurotransmitters will provide insights into the interplay between signaling pathways in the brain (*57*). We are developing multimodal neuroprobes with aptamer-FET sensors for neurotransmitter detection and metal electrodes for electrophysiological recordings. Advances in these directions will provide improve knowledge about fundamental brain function and the etiologies of neurological and neuropsychiatric disorders and are anticipated to accelerate discovery and deployment of improved treatment modalities for psychiatric and neurological disorders.

There are challenges remaining with the neuroprobe technology described. For example, while we have achieved temporal resolution on the order of seconds using aptamer-FET sensors (*21*, *23*), subsecond temporal resolution will enable clearer understanding of the dynamic fluxes of neurochemicals and the information encoded therein. This advance requires the development of additional sensing algorithms and instrumentation. Along these lines, we can tune aptamer-target affinities by altering aptamer stem lengths (*23*) or aptamer-target kinetics by stem destabilization (*58*).

For multiplexed neurotransmitter detection, it will be necessary to functionalize individual FETs on the same neuroprobe with different aptamers. Although we have demonstrated duplexing with two aptamers (*21*), it is challenging to functionalize individual microscale devices that are located close to one another. “Addressable” functionalization strategies include microcontact printing (*59*). Electroactive linkers have been demonstrated (*60*) and are under development for the addressable functionalization of small devices (~10 μm apart) with different aptamers. Moreover, we are developing custom hardware and software designed to monitor multiple FETs at the same time to overcome current instrumentation limitations.

While Si neuroprobes are generally suitable for acute experiments (e.g., hours), for chronic recordings, challenges arise because of immunological responses that occur after longer implantation times. The Young’s modulus mismatch between implanted devices and brain tissue produces inflammatory responses that interfere with biosensing (*51*, *61*). Here, we report on neuroprobes with reduced dimensions (50 μm) and increased flexibility. Developing neuroprobes based on biocompatible and soft materials is an important ongoing research direction (*21*, *31*).

## MATERIALS AND METHODS

### Materials

Prime quality 4-inch Si wafers (boron-doped P-type Si, 0.001 to 0.005 ohm/cm, thickness of 150 or 50 μm) were purchased from Silicon Valley Microelectronics Inc. (Santa Clara, CA, USA). All chemicals were purchased from Sigma-Aldrich Co. (St. Louis, MO), unless otherwise noted below. Oligonucleotides were obtained from Integrated DNA Technologies (Coralville, IA). SYLGARD 184 for fabricating polydimethylsiloxane (PDMS) wells was from Dow Corning Corporation (Midland, MI). Water was deionized before use (18.2 megohms) via a Milli-Q system (Millipore, Billerica, MA).

### Neuroprobe fabrication

Aqueous solutions of 0.1 M indium(III) nitrate hydrate (99.999%) were spin-coated onto Si substrates at 3000 rpm for 30 s. The coated wafers were annealed at 100°C for 10 min and then at 350°C for 4 hours to form continuous In_2_O_3_ films. A photolithography process was applied to define the source and drain electrodes. Electrodes of 10-nm-thick Ti and overlaying 30-nm-thick Au films were fabricated using a CHA solution electron-beam evaporator (CHA Industries Inc., Fremont, CA) under high vacuum (10^−8^ torr) at an evaporation rate of 0.1 nm/s. A thin layer of parylene (~1 μm) was coated on probe surfaces using an SCS Parylene C coating system (Specialty Coating System Inc., Indianapolis, IN), defined photolithography, and then etched by oxygen plasma to expose the FETs on the neuroprobe tips. Another photolithographic treatment was performed to define the probe outlines. Deep reactive ion etching with the Bosch process was used to etch through silicon substrates using a Deep Silicon Etcher III (Plasma-Therm, Fremont, CA).

Probes on wafers or post-release were rinsed in ethanol and dried with N_2_ to clean their surfaces. After cleaning, (3-aminopropyl)triethoxysilane and trimethoxy(propyl)silane (1:9, v/v) were thermally deposited on In_2_O_3_ at 40°C for 1 hour and annealed at 60°C for 10 min. Source and drain interdigitated electrodes were insulated with a self-assembled monolayer by immersing into 1 mM ethanolic solutions of 1-dodecanethiol for 1 hour. Probes were immersed in a 1 mM solution of 3-maleimidobenzoic acid *N*-hydroxysuccinimide ester dissolved in a 1:9 (v/v) mixture of dimethyl sulfoxide and PBS (Gibco, Thermo Fisher Scientific, Waltham, MA) for 30 min. To immobilize aptamers, probes were immersed in a 1 μM solution of thiolated DNA in PBS overnight. Probes were rinsed with deionized water and dried with N_2_ before measurements.

### In vitro and ex vivo experiments

Here, aCSF was prepared as per the detailed protocol in the supplemental information by Zhao *et al.* (*24*). For experiments in aCSF or tissue homogenates, PDMS wells were sealed on top of individual probes (not released from wafers) to hold aCSF and target solutions. The initial solution volume was 40 μl. Serotonin was added in 1 μl aliquots to produce increasing concentrations. After each addition, the solutions in the wells were gently mixed using a micropipette.

For gelatin experiments, a mixture of 10 to 15% gelatin (lot #H219; Mallinckrodt, St. Louis, MO) in aCSF (w/v) (*24*) was prepared first. The mixture was microwaved in 10-s increments (to avoid overheating) for ~1 min until the gelatin was completely dissolved. The clear solution was cast in 1-ml aliquots into 48-well plates (lot #CLS3548; Sigma-Aldrich Co., St. Louis, MO). A mold for holes was templated into the gelatin using a 0.125-inch-diameter metal wire (lot #7667A12; McMaster-Carr Supply Co., Atlanta, GA) for the addition of neurotransmitter solutions to simulate neurotransmitter release in the brain. The gelatin solution was kept at 4°C for ~12 hours. Afterward, the metal mold was taken out carefully using tweezers. The Ag/AgCl gate electrodes for this experiment and the in vivo experiment were constructed of 0.010-inch-diameter Ag wire freshly coated with AgCl (A-M Systems, Sequim, WA) by immersing in a bleach solution (Clorox, Oakland, CA) for 5 min. A neuroprobe and a Ag/AgCl reference electrode were each implanted into a single well containing gelatin using tweezers before measurements. A 2-μl aliquot of 50 μM serotonin was added into each templated well containing gelatin for a final concentration of 100 nM serotonin.

For ex vivo experiments, brain tissue lacking serotonin was obtained from *Tph2* knockout mice (*39*). These mice lack the enzyme (tryptophan hydroxylase 2) needed to synthesize brain serotonin. Most serotonin in the brain is stored in synaptic vesicles. To prepare brain tissue homogenates, cell membranes are disrupted, releasing vesicular serotonin into the homogenates. Hence, the concentration of serotonin in homogenates is much higher than that occurring in vivo in the extracellular space. By using brain tissue from *Tph2*-null mice, we avoided effects of endogenous serotonin in homogenates. Mice were exsanguinated by cardiac perfusion, and brains were cleared of blood containing serotonin synthesized by peripheral tryptophan hydroxylase 1. The brains were then shipped to the University of California, Los Angeles (UCLA) on dry ice from the laboratory of D. Kuhn (Wayne State University, Detroit, MI). Brain tissue was stored at −80°C until use. Brain tissue collection procedures were approved by the Wayne State University Institutional Animal Care and Use Committee.

Brain tissue was homogenized in ice-cold aCSF [1:1 (w/v)] using a VirTis Virsonic 600 ultrasonic cell disruptor (Gardiner, NY) with the microtip set at 4 with 50% duty for 30 to 40 1-s pulses. Commercially available Ag/AgCl reference electrodes (Super Dri-Ref, World Precision Instruments Inc., Sarasota, FL) were placed in the tissue homogenates above the FETs. All FET measurements, including the in vivo measurements described below, were performed using a Keithley 4200A-SCS (Tektronix, Beaverton, OR) semiconductor analyzer. Source-drain current (*I*_DS_) transfer curves were obtained by sweeping the gate voltage (*V*_GS_) from 100 to 350 mV, while maintaining the drain voltage (*V*_DS_) at 10 mV, which took ~5 s for each scan. Calibrated responses were calculated at 300 mV to minimize device-to-device variation, as previously described (*23*).

### In vivo experiments

The Association for Assessment and Accreditation of Laboratory Animal Care International has fully accredited UCLA. All animal care and use met the requirements of the National Institutes of Health *Guide for the Care and Use of Laboratory Animals*, revised 2011. The UCLA Chancellor’s Animal Research Committee (Institutional Animal Care and Use Committee) preapproved all procedures involving animals carried out at UCLA. Mice were generated from a serotonin transporter–deficient lineage on a mixed CD1 × 129S6/SvEv background via heterozygous pairings. Two female serotonin transporter knockout mice (SERT^−/−^) were studied at 4 to 6 months of age. One mouse was used for a pilot study to determine biosensor and stimulating electrode experimental conditions. A second mouse was used to collect the data in Fig. 5. Mice were housed in groups of four to five same-sex siblings per cage until head-bar implantation surgery, after which mice were individually housed. The light-dark cycle was set to 12 hours/12 hours with lights on at 0600. All testing was carried out during the light phase. Food and water were available ad libitum with the exception of experimental testing days.

Surgeries were carried out under aseptic conditions with isoflurane anesthesia on the Kopf Model 1900 Stereotaxic Alignment System (Kopf, Tujunga, CA). Beginning on the day of surgery, we administered the nonsteroidal anti-inflammatory drugs carprofen (subcutaneously daily at 5 mg/kg for 3 days) and ibuprofen (0.25 mg/ml in the drinking water for 14 days), in addition to the antibiotic amoxicillin (0.25 mg/ml) in the drinking water for 14 days. Animals underwent a surgical procedure for head-bar implantation. A rectangular head-bar (9 mm by 7 mm by 0.76 mm; 0.6 g, laser-cut from stainless steel at Fab2Order) for head fixation was attached to each side of the skull by C&B-Metabond (Parkell, Edgewood, NY) (*14*). After surgery, animals recovered for 1 to 3 weeks.

During recovery from head-bar implantation, subjects were trained to acclimate to head fixation by hand for 15 to 30 min per session × 1 to 2 sessions/day for a total 6 to 10 sessions. Afterward, a second surgery to make three craniotomies was carried out 24 to 48 hours ahead of in vivo recordings. A 2.5-mm-wide (medio-lateral) by 1.0-mm-long (anterio-posterior) piece of skull was surgically removed over the brain stem serotonin cell body region (centered at AP −4.48 mm and ML ±0.00 mm from bregma) for the insertion of a stimulating electrode. A 1.5-mm-wide by 1.5-mm-long craniotomy aimed at the right striatum (centered at AP +0.80 mm and ML +0.80 mm from bregma) was made for the insertion of an aptamer-FET neuroprobe. An additional 0.4-mm-diameter hole (centered at AP +2.80 mm and ML −2.00 mm from bregma) was made on the left side of the skull for Ag/AgCl gate electrode implantation.

The dura was left intact over the surgery areas. The craniotomies were sealed with a thin layer of Kwik-Cast and Kwik-Sil (World Precision Instruments, Sarasota, FL). The entire surgery area was then secured with a thin top layer of C&B-Metabond. On the testing day, mice were transferred from their home cages and mounted to a head-fixed stage via their head-bars. Each subject was supported on a Styrofoam ball that served as a treadmill for the subject to engage freely in locomotion.

After a 10-min habituation period, the top layer of C&B-Metabond was carefully removed. The thin layer of Kwik-Cast and Kwik-Sil and the dura above the brain in the craniotomy areas were then removed using ceramic-coated Dumont #5 forceps (Roboz Surgical Instrument Co., Gaithersburg, MD). A Ag/AgCl gate electrode was manually lowered into the designated craniotomy site at 1.5 to 2 mm in depth. An untwisted two-channel tungsten stimulation electrode (Plastics One, Roanoke, VA) was lowered 3.5 mm from the skull level aimed at the serotonin cell body region using a 10-μm precision manual micromanipulator (Narishige International, Amityville, NY). The stimulation electrode tips were 2.0 mm apart.

After another 10-min habituation period, a train of electrical stimulation pulses (biphasic 300 μA × 4 ms at 30 Hz for 5 s) was delivered, which evoked behavioral responses such as freezing, running, shaking, and a notable increase in breathing rate. If no behavioral response was observed, the stimulating electrode was lowered an additional 50 μm per step. Another train of stimulation was delivered with a >5-min interval. This process of locating the stimulating electrode continued until strong stimulation-induced behavioral responses were observed. After positioning, the stimulation electrode (dorso-ventral of −3.5 to −4.5 mm) remained in the same location throughout the experiment.

The source and drain electrodes of the aptamer-FET neuroprobes were connected with wiring using Ag epoxy (Ted Pella, Redding, CA) before functionalization (Fig. 5B) (*25*). The FETs were then functionalized with the serotonin aptamer as described above. Source/drain wires were connected directly to a Keithley 4200A semiconductor analyzer for measurements. An aptamer-FET neuroprobe was lowered to 1.0 mm above the brain surface by hand. A 1-μm precision motorized digital micromanipulator (MP-225; Sutter Instrument, Novato, CA) was then used to lower the probe slowly into the brain. A biphasic, 300-μA, 4-ms, and 30-Hz waveform was then applied for 5 s to the stimulating electrode to evoke serotonin release. Source-drain current (*I*_DS_) transfer curves were obtained by sweeping the gate voltage (*V*_GS_) from 100 to 350 mV while maintaining the drain voltage (*V*_DS_) at 10 mV. Calibrated responses were calculated at 300 mV to minimize device-to-device variation as previously described (*23*). Five baseline calibrated responses were collected before each stimulation. Calibrated responses were collected every 5 s over a period of ca. 60 s after each stimulation (fig. S4).

At each recording depth, two to four stimulation trains were delivered at 5- to 10-min intervals. The neuroprobe was lowered 50 to 150 μm per step for additional testing. Throughout the experiment, sterile saline was used to keep the exposed skull and brain moist. Sweetened condensed milk diluted with drinking water [1:2 (v/v)] was delivered to the subject every 2 to 3 hours using a dropper. The overall health and behavioral responses to stimulation of the mice were closely monitored throughout the experiment. At the end of the experiment, both electrodes and the neuroprobe were removed. Subjects were euthanized, and the brains were prepared for histological verification of the positions of the stimulation and recording paths/sites.

### Data analysis

Data for selectivity testing and gelatin sensing were analyzed by one-way analysis of variance with Dunnett’s or Turkey’s post hoc tests, respectively. Areas under the curves for stimulation data were calculated by trapezoidal integration of seven stimulated serotonin measurements, which defined each stimulation peak. Baselines for integration were determined by a linear fit of five prestimulus points (basal). Data for in vivo responses were analyzed by two-tailed paired *t* tests (GraphPad Prism 7.04, San Diego, CA).

## Supplementary Materials

### This PDF file includes:

Figs. S1 to S4 Tables S1 and S2 References

## Notes

View/request a protocol for this paper from *Bio-protocol*.
