# Supplementary material for: Implantable aptamer–field-effect transistor neuroprobes for in vivo neurotransmitter monitoring
Source: Sci Adv. 2021 Nov 24;7(48):eabj7422. doi: 10.1126/sciadv.abj7422 (PMC8612678; doi:10.1126/sciadv.abj7422)
Supplement: Supplementary file 1 — Figs. S1 to S4 Tables S1 and S2 References [file sciadv.abj7422_sm.pdf]

Science Advances
AAAS

Supplementary Materials for

Implantable aptamer–field-effect transistor neuroprobes for in vivo neurotransmitter monitoring

Chuanzhen Zhao, Kevin M. Cheung, I-Wen Huang, Hongyan Yang, Nako Nakatsuka, Wenfei Liu, Yan Cao, Tianxing Man, Paul S. Weiss, Harold G. Monbouquette, Anne M. Andrews*

*Corresponding author. Email: aandrews@mednet.ucla.edu

Published 24 November 2021, *Sci. Adv.* **7**, eabj7422 (2021)
DOI: 10.1126/sciadv.abj7422

The PDF file includes:

Figs. S1 to S4
Tables S1 and S2
References

Other Supplementary Material for this manuscript includes the following:

Movie S1

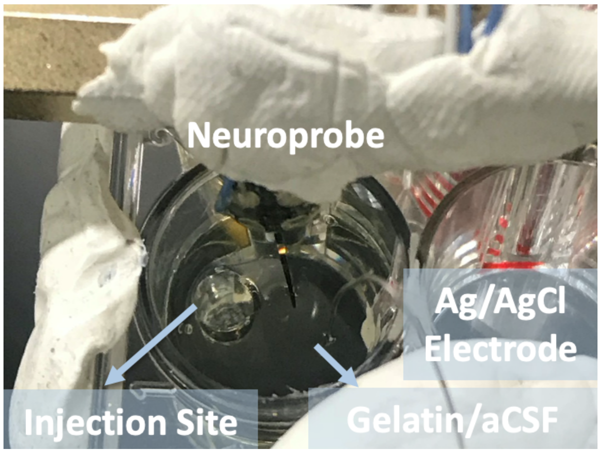

Figure S1. Gelatin brain mimic experiment. Photograph of the experimental set-up where individual wells in a 48-well plate (~11 mm in diameter) were used to mimic the size of a mouse brain (~1 cm in diameter). A hole was templated in each gelatin for the addition of serotonin during the experiment to simulate neurotransmitter release and diffusion to the recording site on the neuroprobe. The hole for serotonin delivery was ~2 mm from the recording site. Photo Credit: Chuanzhen Zhao, UCLA.

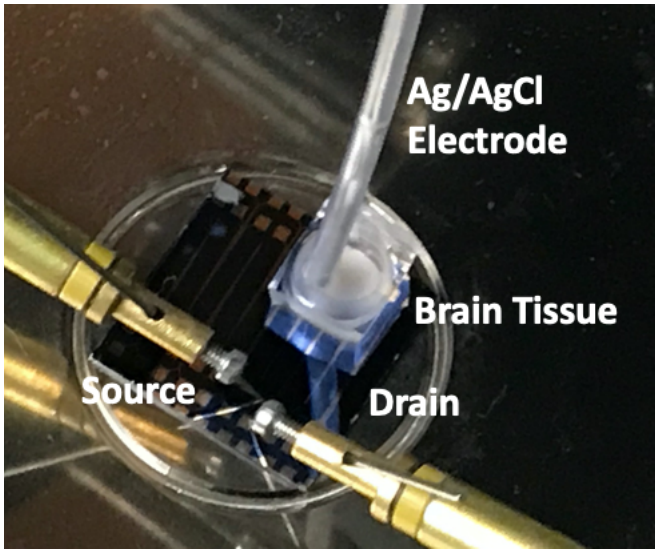

Figure S2. Photograph of the *ex vivo* setup. Brain tissue homogenate was added to a polydimethylsiloxane well with a Ag/AgCl electrode. Photo Credit: Chuanzhen Zhao, UCLA.

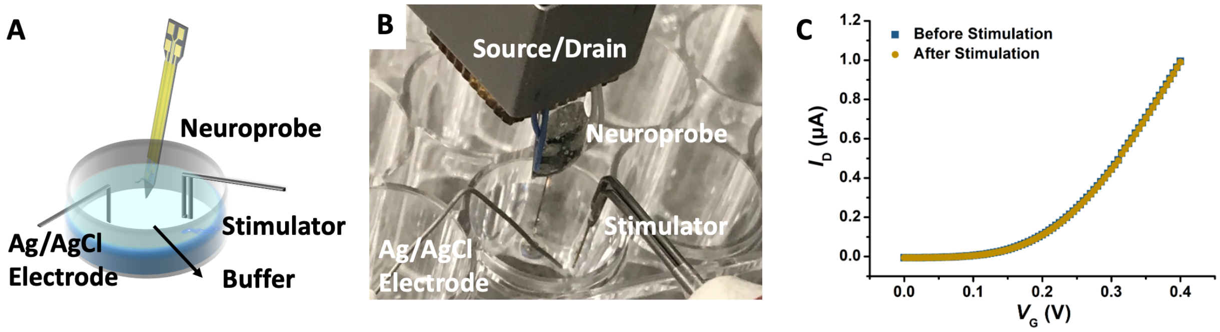

Figure S3. Effect of electrical stimulation on neuroprobe stability. (A) Schematic and **(B)** photograph of the experimental set-up, where a neuroprobe was placed into phosphate-buffered saline with a stimulating electrode. **(C)** Representative I_{DS}-V_{GS} sweeps immediately before and after electrical stimulation (biphasic pulses of 300 μA, 4 ms at 30 Hz for 5 s) showing overlap of the two curves. Photo Credit: Chuanzhen Zhao, UCLA.

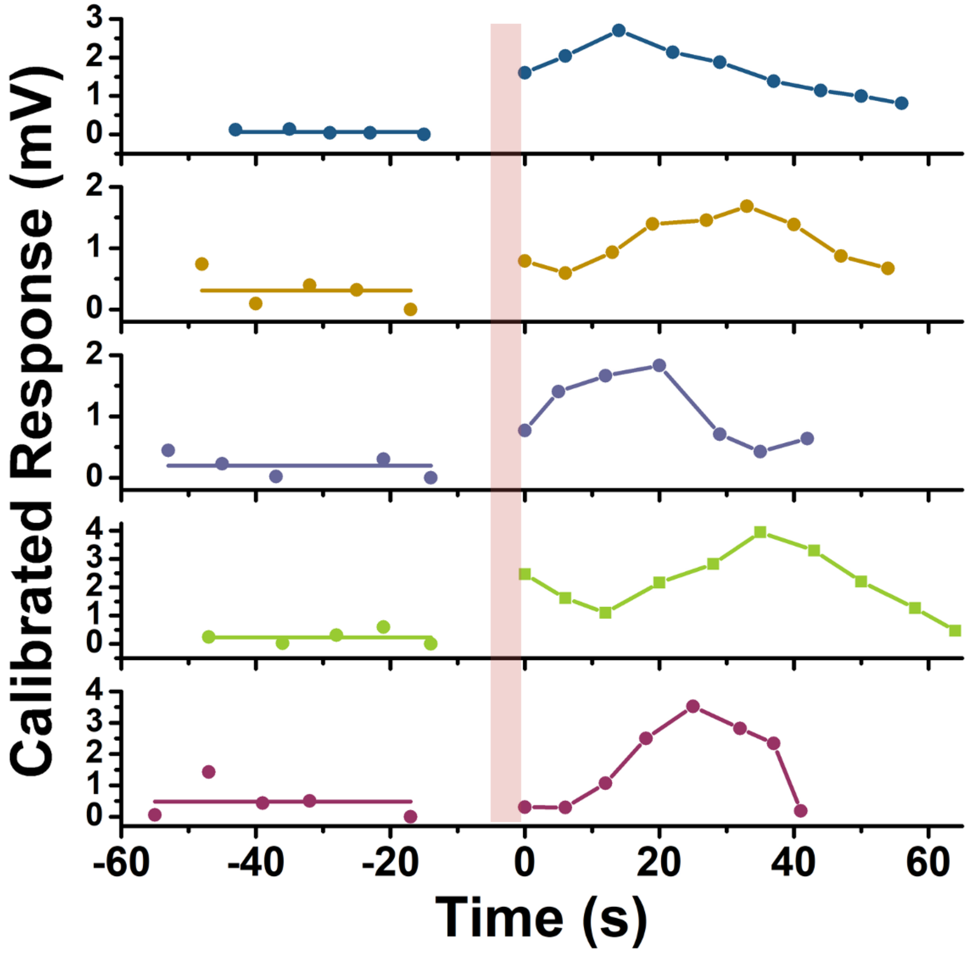

Figure S4. Real-time *in vivo* responses. Five representative *in vivo* serotonin response curves before and after electrical stimulation (biphasic, 300 μA, 4 ms, at 30 Hz for 5 s, shown in pink) from the same awake mouse.

Table S1. Summary of statistics. Reponses from biosensors were analyzed by one-way analysis of variance (ANOVA) or paired *t*-tests.

**Type**	**Figure**	**ANOVA**	**Multiple Comparisons**
Selectivity	Fig. 3G	F (6,15) = 14.31<br>$P < 0.0001$	P <0.01
Gelatin	Fig. 4B	F (2,3) = 17.76<br>$P < 0.05$	0 min *vs.* 3 min, Not signficant<br>0 min *vs.* 12 min, P < 0.05<br>3 min *vs.* 12 min, P < 0.05

**Type**	**Figure**	**Paired *t*-Test**	**t, df**
Average of response	Fig. 5E	$P < 0.01$<br>Two-tailed P value	t = 5.893<br>df = 4
Area under the curve	Fig. 5F	$P < 0.01$<br>Two-tailed P value	t = 5.041<br>df = 4

Table S2. Comparison of *in vivo* neurochemical monitoring techniques.

Sensing Method	Advantages	Limitations
**Aptamer FET (*21-24*)**	1. Very high sensitivity (fM)<br>2. Very high selectivity imparted by counter-selected aptamers<br>3. Very high spatial resolution (FETs can be nm, *e.g.*, carbon nanotubes)<br>4. Can detect all species regardless of target charge, availability of catabolic enzyme, or electroactivity<br>5. Label free<br>6. Can be multiplexed	1. Temporal resolution (s); not yet optimized
**Fast scan cyclic voltammetry (FSCV) (*36, 37, 62-67*)**	1. High sensitivity (nM)<br>2. High temporal resolution (100 ms)<br>3. High spatial resolution (smallest sensors are 5-7 μm)<br>4. Label free	1. Limited to electroactive species<br>2. Limited selectivity<br>3. Limited multiplexing
**Microdialysis with HPLC (*34, 40, 68-73*)**	1. Very high sensitivity (fM)<br>2. Very high selectivity imparted by separations<br>3. Can detect all species regardless of target charge, availability of catabolic enzyme, or electroactivity<br>4. Label free<br>5. Can be multiplexed	1. Limited temporal resolution (min)<br>2. Limited spatial resolution (microdialysis probes are >100 μm in width)<br>3. Expensive analytical instrumentation needed
**Fluorescence imaging (genetically encoded) (*74-80*)**	1. Very high sensitivity<br>2. Very high temporal resolution (ms)<br>3. Very high spatial resolution (single neuron/bouton resolution with two-photon microscopy)	1. Requirement for genetic encoding<br>2. For high spatial resolution, expensive microscope needed<br>3. Limited multiplexing

Aptamer field-effect transistor biosensor design consideration

To design a transistor biosensor with high sensitivity, a high transconductance is preferred. There are mainly three parameters to be considered to increase the transconductance (g_m): semiconductor mobility (μ), capacitance (C), and channel width (W).

$$g_m = \frac{I_{out}}{V_{in}} = \mu \cdot C \cdot \frac{W}{L} \cdot (V_g - V_T)$$

Indium oxide was chosen as the semiconductor due to its high mobility compared with other materials (*e.g.*, organic semiconductors) (*28-31*). In our system, transistors were operated in physiological environments with high dielectric constants (*e.g.*, dielectric constant ~80 for phosphate buffer), which maximizes the capacitance. To increase the channel width, we used interdigitated electrodes to increase the transconductance.

Calculation method of calibrated response

The calibrated response is used to minimize device-to-device variations (*23, 35*). In our measurement setup, the drain voltage (V_d) was biased at 10 mV while the we sweep the gate voltages (V_g). The absolute sensor response (ΔI_d), which takes into account baseline subtraction, is divided by the change in source-drain current with voltage sweep (dI_d/dV_g). The reported calibrated response was calculated at V_g = 300 mV when the FET is turned on. The equation for this calculation is:

$$\boldsymbol{Calibrated\ response\ (\Delta V)} = \frac{\boldsymbol{\Delta I_d}}{\boldsymbol{dI_d}/\boldsymbol{dV_g}}$$

REFERENCES AND NOTES

1. A. P. Alivisatos, A. M. Andrews, E. S. Boyden, M. Chun, G. M. Church, K. Deisseroth, J. P. Donoghue, S. E. Fraser, J. Lippincott-Schwartz, L. L. Looger, S. Masmanidis, P. L. McEuen, A. V. Nurmikko, H. Park, D. S. Peterka, C. Reid, M. L. Roukes, A. Scherer, M. Schnitzer, T. J. Sejnowski, K. L. Shepard, D. Tsao, G. Turrigiano, P. S. Weiss, C. Xu, R. Yuste, X. Zhuang, Nanotools for neuroscience and brain activity mapping. *ACS Nano* **7**, 1850–1866 (2013).

2. S. C. Altieri, H. Yang, H. J. O'Brien, H. M. Redwine, D. Senturk, J. G. Hensler, A. M. Andrews, Perinatal *vs* genetic programming of serotonin states associated with anxiety. *Neuropsychopharmacology* **40**, 1456–1470 (2015).

3. M. Malvaez, C. Shieh, M. D. Murphy, V. Y. Greenfield, K. M. Wassum, Distinct cortical-amygdala projections drive reward value encoding and retrieval. *Nat. Neurosci.* **22**, 762–769 (2019).

4. N. Dolensek, D. A. Gehrlach, A. S. Klein, N. Gogolla, Facial expressions of emotion states and their neuronal correlates in mice. *Science* **368**, 89–94 (2020).

5. K. Lee, L. D. Claar, A. Hachisuka, K. I. Bakhurin, J. Nguyen, J. M. Trott, J. L. Gill, S. C. Masmanidis, Temporally restricted dopaminergic control of reward-conditioned movements. *Nat. Neurosci.* **23**, 209–216 (2020).

6. U. Topalovic, Z. M. Aghajan, D. Villaroman, S. Hiller, L. Christov-Moore, T. J. Wishard, M. Stangl, N. R. Hasulak, C. S. Inman, T. A. Fields, V. R. Rao, D. Eliashiv, I. Fried, N. Suthana, Wireless programmable recording and stimulation of deep brain activity in freely moving humans. *Neuron* **108**, 322–334.e9 (2020).

7. J. P. Seymour, F. Wu, K. D. Wise, E. Yoon, State-of-the-art MEMS and microsystem tools for brain research. *Microsyst. Nanoeng.* **3**, 16066 (2017).

8. S. M. Won, E. Song, J. Zhao, J. Li, J. Rivnay, J. A. Rogers, Recent advances in materials, devices, and systems for neural interfaces. *Adv. Mater.* **30**, e1800534 (2018).

9. G. A. Woods, N. J. Rommelfanger, G. Hong, Bioinspired materials for *in vivo* bioelectronic neural interfaces. *Matter* **3**, 1087–1113 (2020).

10. R. Chen, A. Canales, P. Anikeeva, Neural recording and modulation technologies. *Nat. Rev. Mater.* **2**, 16093 (2017).

11. M. D. Ferro, N. A. Melosh, Electronic and ionic materials for neurointerfaces. *Adv. Funct. Mater.* **28**, 1704335 (2018).

12. J. Viventi, D. H. Kim, L. Vigeland, E. S. Frechette, J. A. Blanco, Y. S. Kim, A. E. Avrin, V. R. Tiruvadi, S. W. Hwang, A. C. Vanleer, D. F. Wulsin, K. Davis, C. E. Gelber, L. Palmer, J. Van der Spiegel, J. Wu, J. Xiao, Y. Huang, D. Contreras, J. A. Rogers, B. Litt, Flexible, foldable, actively multiplexed, high-density electrode array for mapping brain activity *in vivo*. *Nat. Neurosci.* **14**, 1599–1605 (2011).

13. N. Vachicouras, O. Tarabichi, V. V. Kanumuri, C. M. Tringides, J. Macron, F. Fallegger, Y. Thenaisie, L. Epprecht, S. McInturff, A. A. Qureshi, V. Paggi, M. W. Kuklinski, M. C. Brown, D. J. Lee, S. P. Lacour, Microstructured thin-film electrode technology enables proof of concept of scalable, soft auditory brainstem implants. *Sci. Transl. Med.* **11**, eaax9487 (2019).

14. J. Du, T. J. Blanche, R. R. Harrison, H. A. Lester, S. C. Masmanidis, Multiplexed, high density electrophysiology with nanofabricated neural probes. *PLOS ONE* **6**, e26204 (2011).

15. G. Rios, E. V. Lubenov, D. Chi, M. L. Roukes, A. G. Siapas, Nanofabricated neural probes for dense 3-D recordings of brain activity. *Nano Lett.* **16**, 6857–6862 (2016).

16. J. J. Jun, N. A. Steinmetz, J. H. Siegle, D. J. Denman, M. Bauza, B. Barbarits, A. K. Lee, C. A. Anastassiou, A. Andrei, C. Aydin, M. Barbic, T. J. Blanche, V. Bonin, J. Couto, B. Dutta, S. L. Gratiy, D. A. Gutnisky, M. Hausser, B. Karsh, P. Ledochowitsch, C. M. Lopez, C. Mitelut, S. Musa, M. Okun, M. Pachitariu, J. Putzeys, P. D. Rich, C. Rossant, W. L. Sun, K. Svoboda, M. Carandini, K. D. Harris, C. Koch, J. O'Keefe, T. D. Harris, Fully integrated silicon probes for high-density recording of neural activity. *Nature* **551**, 232–236 (2017).

17. N. Driscoll, A. G. Richardson, K. Maleski, B. Anasori, O. Adewole, P. Lelyukh, L. Escobedo, D. K. Cullen, T. H. Lucas, Y. Gogotsi, F. Vitale, Two-dimensional Ti_3C_2 MXene for high-resolution neural interfaces. *ACS Nano* **12**, 10419–10429 (2018).

18. Y. Jiang, X. Li, B. Liu, J. Yi, Y. Fang, F. Shi, X. Gao, E. Sudzilovsky, R. Parameswaran, K. Koehler, V. Nair, J. Yue, K. Guo, H.-M. Tsai, G. Freyermuth, R. C. S. Wong, C.-M. Kao, C.-T. Chen, A. W. Nicholls, X. Wu, G. M. G. Shepherd, B. Tian, Rational design of silicon structures for optically controlled multiscale biointerfaces. *Nat. Biomed. Eng.* **2**, 508–521 (2018).

19. G. Hong, T. M. Fu, M. Qiao, R. D. Viveros, X. Yang, T. Zhou, J. M. Lee, H. G. Park, J. R. Sanes, C. M. Lieber, A method for single-neuron chronic recording from the retina in awake mice. *Science* **360**, 1447–1451 (2018).

20. J. E. Chung, H. R. Joo, J. L. Fan, D. F. Liu, A. H. Barnett, S. Chen, C. Geaghan-Breiner, M. P. Karlsson, M. Karlsson, K. Y. Lee, H. Liang, J. F. Magland, J. A. Pebbles, A. C. Tooker, L. F. Greengard, V. M. Tolosa, L. M. Frank, High-density, long-lasting, and multi-region electrophysiological recordings using polymer electrode arrays. *Neuron* **101**, 21–31.e5 (2019).

21. Q. Liu, C. Zhao, M. Chen, Y. Liu, Z. Zhao, F. Wu, Z. Li, P. S. Weiss, A. M. Andrews, C. Zhou, Flexible multiplexed In_2O_3 nanoribbon aptamer-field-effect transistors for biosensing. *iScience* **23**, 101469 (2020).

22. K. M. Cheung, K. A. Yang, N. Nakatsuka, C. Zhao, M. Ye, M. E. Jung, H. Yang, P. S. Weiss, M. N. Stojanović, A. M. Andrews, Phenylalanine monitoring *via* aptamer-field-effect transistor sensors. *ACS Sens.* **4**, 3308–3317 (2019).

23. N. Nakatsuka, K. A. Yang, J. M. Abendroth, K. M. Cheung, X. Xu, H. Yang, C. Zhao, B. Zhu, Y. S. Rim, Y. Yang, P. S. Weiss, M. N. Stojanović, A. M. Andrews, Aptamer-field-effect transistors overcome Debye length limitations for small-molecule sensing. *Science* **362**, 319–324 (2018).

24. C. Zhao, Q. Liu, K. M. Cheung, W. Liu, Q. Yang, X. Xu, T. Man, P. S. Weiss, C. Zhou, A. M. Andrews, Narrower nanoribbon biosensors fabricated by chemical lift-off lithography show higher sensitivity. *ACS Nano* **15**, 904–915 (2021).

25. K. M. Wassum, V. M. Tolosa, J. Wang, E. Walker, H. G. Monbouquette, N. T. Maidment, Silicon wafer-based platinum microelectrode array biosensor for near real-time measurement of glutamate *in vivo*. *Sensors* **8**, 5023–5036 (2008).

26. L. C. Moreaux, D. Yatsenko, W. D. Sacher, J. Choi, C. Lee, N. J. Kubat, R. J. Cotton, E. S. Boyden, M. Z. Lin, L. Tian, A. S. Tolias, J. K. S. Poon, K. L. Shepard, M. L. Roukes, Integrated neurophotonics: Toward dense volumetric interrogation of brain circuit activity-at depth and in real time. *Neuron* **108**, 66–92 (2020).

27. B. Wang, X. Wen, Y. Cao, S. Huang, H. A. Lam, T. L. Liu, P. S. Chung, H. G. Monbouquette, P. Y. Chiou, N. T. Maidment, An implantable multifunctional neural microprobe for simultaneous multi-analyte sensing and chemical delivery. *Lab Chip* **20**, 1390–1397 (2020).

28. H. Chen, Y. S. Rim, I. C. Wang, C. Li, B. Zhu, M. Sun, M. S. Goorsky, X. He, Y. Yang, Quasi-two-dimensional metal oxide semiconductors based ultrasensitive potentiometric biosensors. *ACS Nano* **11**, 4710–4718 (2017).

29. N. Aroonyadet, X. Wang, Y. Song, H. Chen, R. J. Cote, M. E. Thompson, R. H. Datar, C. Zhou, Highly scalable, uniform, and sensitive biosensors based on top-down indium oxide nanoribbons and electronic enzyme-linked immunosorbent assay. *Nano Lett.* **15**, 1943–1951 (2015).

30. J. Kim, Y. S. Rim, H. Chen, H. H. Cao, N. Nakatsuka, H. L. Hinton, C. Zhao, A. M. Andrews, Y. Yang, P. S. Weiss, Fabrication of high-performance ultrathin In_2O_3 film field-effect transistors and biosensors using chemical lift-off lithography. *ACS Nano* **9**, 4572–4582 (2015).

31. Y. S. Rim, S. H. Bae, H. Chen, J. L. Yang, J. Kim, A. M. Andrews, P. S. Weiss, Y. Yang, H. R. Tseng, Printable ultrathin metal oxide semiconductor-based conformal biosensors. *ACS Nano* **9**, 12174–12181 (2015).

32. B. J. Kim, J. T. Kuo, S. A. Hara, C. D. Lee, L. Yu, C. A. Gutierrez, T. Q. Hoang, V. Pikov, E. Meng, 3D parylene sheath neural probe for chronic recordings. *J. Neural Eng.* **10**, 045002 (2013).

33. C. Metallo, R. D. White, B. A. Trimmer, Flexible parylene-based microelectrode arrays for high resolution EMG recordings in freely moving small animals. *J. Neurosci. Methods* **195**, 176–184 (2011).

34. H. Yang, A. B. Thompson, B. J. McIntosh, S. C. Altieri, A. M. Andrews, Physiologically relevant changes in serotonin resolved by fast microdialysis. *ACS Chem. Nerosci.* **4**, 790–798 (2013).

35. F. N. Ishikawa, M. Curreli, H. K. Chang, P. C. Chen, R. Zhang, R. J. Cote, M. E. Thompson, C. Zhou, A calibration method for nanowire biosensors to suppress device-to-device variation. *ACS Nano* **3**, 3969–3976 (2009).

36. H. Shin, Y. Oh, C. Park, Y. Kang, H. U. Cho, C. D. Blaha, K. E. Bennet, M. L. Heien, I. Y. Kim, K. H. Lee, D. P. Jang, Sensitive and selective measurement of serotonin in vivo using fast cyclic square-wave voltammetry. *Anal. Chem.* **92**, 774–781 (2020).

37. Y. Oh, M. L. Heien, C. Park, Y. M. Kang, J. Kim, S. L. Boschen, H. Shin, H. U. Cho, C. D. Blaha, K. E. Bennet, H. K. Lee, S. J. Jung, I. Y. Kim, K. H. Lee, D. P. Jang, Tracking tonic dopamine levels *in vivo* using multiple cyclic square wave voltammetry. *Biosens. Bioelectron.* **121**, 174–182 (2018).

38. K. Scida, K. W. Plaxco, B. G. Jamieson, High frequency, real-time neurochemical and neuropharmacological measurements *in situ* in the living body. *Transl. Res.* **213**, 50–66 (2019).

39. M. A. Kandadai, J. L. Raymond, G. J. Shaw, Comparison of electrical conductivities of various brain phantom gels: Developing a 'brain gel model'. *Mater. Sci. Eng. C* **32**, 2664–2667 (2012).

40. H. Yang, M. M. Sampson, D. Senturk, A. M. Andrews, Sex- and SERT-mediated differences in stimulated serotonin revealed by fast microdialysis. *ACS Chem. Nerosci.* **6**, 1487–1501 (2015).

41. T. A. Mathews, D. E. Fedele, F. M. Coppelli, A. M. Avila, D. L. Murphy, A. M. Andrews, Gene dose-dependent alterations in extraneuronal serotonin but not dopamine in mice with reduced serotonin transporter expression. *J. Neurosci. Methods* **140**, 169–181 (2004).

42. A. Abdalla, C. W. Atcherley, P. Pathirathna, S. Samaranayake, B. Qiang, E. Pena, S. L. Morgan, M. L. Heien, P. Hashemi, I*n vivo* ambient serotonin measurements at carbon-fiber microelectrodes. *Anal. Chem.* **89**, 9703–9711 (2017).

43. M. Angoa-Perez, M. J. Kane, D. I. Briggs, N. Herrera-Mundo, C. E. Sykes, D. M. Francescutti, D. M. Kuhn, Mice genetically depleted of brain serotonin do not display a depression-like behavioral phenotype. *ACS Chem. Nerosci.* **5**, 908–919 (2014).

44. N. T. Rodeberg, S. G. Sandberg, J. A. Johnson, P. E. Phillips, R. M. Wightman, Hitchhiker's guide to voltammetry: Acute and chronic electrodes for *in vivo* fast-scan cyclic voltammetry. *ACS Chem. Nerosci.* **8**, 221–234 (2017).

45. K. I. Bakhurin, V. Mac, P. Golshani, S. C. Masmanidis, Temporal correlations among functionally specialized striatal neural ensembles in reward-conditioned mice. *J. Neurophysiol.* **115**, 1521–1532 (2016).

46. F. C. Zhou, K.-P. Lesch, D. L. Murphy, Serotonin uptake into dopamine neurons *via* dopamine transporters: A compensatory alternative. *Brain Res.* **942**, 109–119 (2002).

47. M. Zhou, K. Engel, J. Wang, Evidence for significant contribution of a newly identified monoamine transporter (PMAT) to serotonin uptake in the human brain. *Biochem. Pharmacol.* **73**, 147–154 (2007).

48. L. C. Daws, Unfaithful neurotransmitter transporters: Focus on serotonin uptake and implications for antidepressant efficacy. *Pharmacol. Ther.* **121**, 89–99 (2009).

49. X. A. Perez, A. M. Andrews, Chronoamperometry to determine differential reductions in uptake in brain synaptosomes from serotonin transporter knockout mice. *Anal. Chem.* **77**, 818–826 (2005).

50. S. Montanez, W. A. Owens, G. G. Gould, D. L. Murphy, L. C. Daws, Exaggerated effect of fluvoxamine in heterozygote serotonin transporter knockout mice. *J. Neurochem.* **86**, 210–219 (2003).

51. T. D. Kozai, A. S. Jaquins-Gerstl, A. L. Vazquez, A. C. Michael, X. T. Cui, Brain tissue responses to neural implants impact signal sensitivity and intervention strategies. *ACS Chem. Nerosci.* **6**, 48–67 (2015).

52. M. Lee, H. J. Shim, C. Choi, D. H. Kim, Soft high-resolution neural interfacing probes: Materials and design approaches. *Nano Lett.* **19**, 2741–2749 (2019).

53. J. Shi, Y. Fang, Flexible and implantable microelectrodes for chronically stable neural interfaces. *Adv. Mater.* **31**, e1804895 (2019).

54. J. Rivnay, H. Wang, L. Fenno, K. Deisseroth, G. G. Malliaras, Next-generation probes, particles, and proteins for neural interfacing. *Sci. Adv.* **3**, e1601649 (2017).

55. J. G. Bruno, M. P. Carrillo, T. Phillips, B. King, Development of DNA aptamers for cytochemical detection of acetylcholine. *In Vitro Cell. Dev. Biol. Anim.* **44**, 63–72 (2008).

56. T. Mairal Lerga, M. Jauset-Rubio, V. Skouridou, A. S. Bashammakh, M. S. El-Shahawi, A. O. Alyoubi, C. K. O'Sullivan, High affinity aptamer for the detection of the biogenic amine histamine. *Anal. Chem.* **91**, 7104–7111 (2019).

57. V. Niederkofler, T. E. Asher, S. M. Dymecki, Functional interplay between dopaminergic and serotonergic neuronal systems during development and adulthood. *ACS Chem. Nerosci.* **6**, 1055–1070 (2015).

58. B. D. Wilson, A. A. Hariri, I. A. P. Thompson, M. Eisenstein, H. T. Soh, Independent control of the thermodynamic and kinetic properties of aptamer switches. *Nat. Commun.* **10**, 5079 (2019).

59. B. Wang, B. Koo, L. W. Huang, H. G. Monbouquette, Microbiosensor fabrication by polydimethylsiloxane stamping for combined sensing of glucose and choline. *Analyst* **143**, 5008–5013 (2018).

60. M. Curreli, C. Li, Y. Sun, B. Lei, M. A. Gundersen, M. E. Thompson, C. Zhou, Selective functionalization of In_2O_3 nanowire mat devices for biosensing applications. *J. Am. Chem. Soc.* **127**, 6922–6923 (2005).

61. Z. J. Du, C. L. Kolarcik, T. D. Y. Kozai, S. D. Luebben, S. A. Sapp, X. S. Zheng, J. A. Nabity, X. T. Cui, Ultrasoft microwire neural electrodes improve chronic tissue integration. *Acta Biomater.* **53**, 46–58 (2017).

62. M. L. Heien, M. A. Johnson, R. M. Wightman, Resolving neurotransmitters detected by fast-scan cyclic voltammetry. *Anal. Chem.* **76**, 5697–5704 (2004).

63. M. K. Zachek, J. Park, P. Takmakov, R. M. Wightman, G. S. McCarty, Microfabricated FSCV-compatible microelectrode array for real-time monitoring of heterogeneous dopamine release. *Analyst* **135**, 1556–1563 (2010).

64. S. B. Flagel, J. J. Clark, T. E. Robinson, L. Mayo, A. Czuj, I. Willuhn, C. A. Akers, S. M. Clinton, P. E. Phillips, H. Akil, A selective role for dopamine in stimulus–reward learning. *Nature* **469**, 53–57 (2011).

65. Y. S. Singh, L. E. Sawarynski, P. D. Dabiri, W. R. Choi, A. M. Andrews, Head-to-head comparisons of carbon fiber microelectrode coatings for sensitive and selective neurotransmitter detection by voltammetry. *Anal. Chem.* **83**, 6658–6666 (2011).

66. C. Park, Y. Oh, H. Shin, J. Kim, Y. Kang, J. Sim, H. U. Cho, H. K. Lee, S. J. Jung, C. D. Blaha, K. E. Bennet, M. L. Heien, K. H. Lee, I. Y. Kim, D. P. Jang, Fast cyclic square-wave voltammetry to enhance neurotransmitter selectivity and sensitivity. *Anal. Chem.* **90**, 13348–13355 (2018).

67. B. J. Venton, Q. Cao, Fundamentals of fast-scan cyclic voltammetry for dopamine detection. *Analyst* **145**, 1158–1168 (2020).

68. K. N. Schultz, R. T. Kennedy, Time-resolved microdialysis for *in vivo* neurochemical measurements and other applications. *Annu. Rev. Anal. Chem.* **1**, 627–661 (2008).

69. Y. Liu, J. Zhang, X. Xu, M. K. Zhao, A. M. Andrews, S. G. Weber, Capillary ultrahigh performance liquid chromatography with elevated temperature for sub-one minute separations of basal serotonin in submicroliter brain microdialysate samples. *Anal. Chem.* **82**, 9611–9616 (2010).

70. O. S. Mabrouk, Q. Li, P. Song, R. T. Kennedy, Microdialysis and mass spectrometric monitoring of dopamine and enkephalins in the globus pallidus reveal reciprocal interactions that regulate movement. *J. Neurochem.* **118**, 24–33 (2011).

71. R. T. Kennedy, Emerging trends in *in vivo* neurochemical monitoring by microdialysis. *Curr. Opin. Chem. Biol.* **17**, 860–867 (2013).

72. J. Zhang, A. Jaquins-Gerstl, K. M. Nesbitt, S. C. Rutan, A. C. Michael, S. G. Weber, *In vivo* monitoring of serotonin in the striatum of freely moving rats with one minute temporal resolution by online

microdialysis-capillary high-performance liquid chromatography at elevated temperature and pressure. *Anal. Chem.* **85**, 9889–9897 (2013).

73. K. T. Ngo, E. L. Varner, A. C. Michael, S. G. Weber, Monitoring dopamine responses to potassium ion and nomifensine by *in vivo* microdialysis with online liquid chromatography at one-minute resolution. *ACS Chem. Nerosci.* **8**, 329–338 (2017).

74. A. Muller, V. Joseph, P. A. Slesinger, D. Kleinfeld, Cell-based reporters reveal *in vivo* dynamics of dopamine and norepinephrine release in murine cortex. *Nat. Methods* **11**, 1245–1252 (2014).

75. R. Liang, G. J. Broussard, L. Tian, Imaging chemical neurotransmission with genetically encoded fluorescent sensors. *ACS Chem. Nerosci.* **6**, 84–93 (2015).

76. K. S. Girven, D. R. Sparta, Probing deep brain circuitry: New advances in *in vivo* calcium measurement strategies. *ACS Chem. Nerosci.* **8**, 243–251 (2017).

77. T. Patriarchi, J. R. Cho, K. Merten, M. W. Howe, A. Marley, W. H. Xiong, R. W. Folk, G. J. Broussard, R. Liang, M. J. Jang, H. Zhong, D. Dombeck, M. von Zastrow, A. Nimmerjahn, V. Gradinaru, J. T. Williams, L. Tian, Ultrafast neuronal imaging of dopamine dynamics with designed genetically encoded sensors. *Science* **360**, eaat4422 (2018).

78. F. Sun, J. Zeng, M. Jing, J. Zhou, J. Feng, S. F. Owen, Y. Luo, F. Li, H. Wang, T. Yamaguchi, Z. Yong, Y. Gao, W. Peng, L. Wang, S. Zhang, J. Du, D. Lin, M. Xu, A. C. Kreitzer, G. Cui, Y. Li, A genetically encoded fluorescent sensor enables rapid and specific detection of dopamine in flies, fish, and mice. *Cell* **174**, 481–496.e19 (2018).

79. E. K. Unger, J. P. Keller, M. Altermatt, R. Liang, A. Matsui, C. Dong, O. J. Hon, Z. Yao, J. Sun, S. Banala, M. E. Flanigan, D. A. Jaffe, S. Hartanto, J. Carlen, G. O. Mizuno, P. M. Borden, A. V. Shivange, L. P. Cameron, S. Sinning, S. M. Underhill, D. E. Olson, S. G. Amara, D. Temple Lang, G. Rudnick, J. S. Marvin, L. D. Lavis, H. A. Lester, V. A. Alvarez, A. J. Fisher, J. A. Prescher, T. L. Kash, V. Yarov-Yarovoy, V. Gradinaru, L. L. Looger, L. Tian, Directed evolution of a selective and sensitive serotonin sensor *via* machine learning. *Cell* **183**, 1986–2002.e26 (2020).

80. J. Wan, W. Peng, X. Li, T. Qian, K. Song, J. Zeng, F. Deng, S. Hao, J. Feng, P. Zhang, Y. Zhang, J. Zou, S. Pan, M. Shin, B. J. Venton, J. J. Zhu, M. Jing, M. Xu, Y. Li, A genetically encoded sensor for measuring serotonin dynamics. *Nat. Neurosci.* **24**, 746–752 (2021).
